# Metabolism Interactions Promote the Overall Functioning of the Episymbiotic Chemosynthetic Community of Shinkaia crosnieri of Cold Seeps

**DOI:** 10.1128/msystems.00320-22

**Published:** 2022-08-08

**Authors:** Zheng Xu, Minxiao Wang, Huan Zhang, Wanying He, Lei Cao, Chao Lian, Zhaoshan Zhong, Hao Wang, Lulu Fu, Xin Zhang, Chaolun Li

**Affiliations:** a CAS Key Laboratory of Marine Ecology and Environmental Sciences, Institute of Oceanology, Chinese Academy of Sciences, Qingdao, China; b Center of Deep Sea Research, Institute of Oceanology, Chinese Academy of Sciences, Qingdao, China; c Laboratory for Marine Ecology and Environmental Science, Qingdao National Laboratory for Marine Science and Technology, Qingdao, China; d Key Lab of Marine Geology and Environment, Institute of Oceanology, Chinese Academy of Sciences, Qingdao, China; e Center for Ocean Mega-Science, Chinese Academy of Sciences, Qingdao, China; f University of Chinese Academy of Sciences, Beijing, China; Max Planck Institute for Marine Microbiology

**Keywords:** chemosynthesis, episymbiont, interaction, cold seep, adaptation

## Abstract

Remarkably diverse bacteria have been observed as biofilm aggregates on the surface of deep-sea invertebrates that support the growth of hosts through chemosynthetic carbon fixation. Growing evidence also indicates that community-wide interactions, and especially cooperation among symbionts, contribute to overall community productivity. Here, metagenome-guided metatranscriptomic and metabolic analyses were conducted to investigate the taxonomic composition, functions, and potential interactions of symbionts dwelling on the seta of Shinkaia crosnieri lobsters in a methane cold seep. *Methylococcales* and *Thiotrichales* dominated the community, followed by the *Campylobacteriales*, *Nitrosococcales*, *Flavobacteriales*, and *Chitinophagales* Metabolic interactions may be common among the episymbionts since many separate taxon genomes encoded complementary genes within metabolic pathways. Specifically, *Thiotrichales* could contribute to detoxification of hydroxylamine that is a metabolic by-product of *Methylococcales*. Further, *Nitrosococcales* may rely on methanol leaked from *Methylococcales* cells that efficiently oxidize methane. Elemental sulfur may also serve as a community good that enhances sulfur utilization that benefits the overall community, as evidenced by confocal Raman microscopy. Stable intermediates may connect symbiont metabolic activities in cyclical oxic-hypoxic fluctuating environments, which then enhance overall community functioning. This hypothesis was partially confirmed via *in situ* experiments. These results highlight the importance of microbe-microbe interactions in symbiosis and deep-sea adaptation.

**IMPORTANCE** Symbioses between chemosynthetic bacteria and marine invertebrates are common in deep-sea chemosynthetic ecosystems and are considered critical foundations for deep-sea colonization. Episymbiotic microorganisms tend to form condensed biofilms that may facilitate metabolite sharing among biofilm populations. However, the prevalence of metabolic interactions among deep-sea episymbionts and their contributions to deep-sea adaptations are not well understood due to sampling and cultivation difficulties associated with deep-sea environments. Here, we investigated metabolic interactions among the episymbionts of *Shinkaia crosnieri*, a dominant chemosynthetic ecosystem lobster species in the Northwest Pacific Ocean. Meta-omics characterizations were conducted alongside *in situ* experiments to validate interaction hypotheses. Furthermore, imaging analysis was conducted, including electron microscopy, fluorescent *in situ* hybridization (FISH), and confocal Raman microscopy (CRM), to provide direct evidence of metabolic interactions. The results support the Black Queen Hypothesis, wherein leaked public goods are shared among cohabitating microorganisms to enhance the overall adaptability of the community via cooperation.

## INTRODUCTION

Deep-sea chemosynthetic ecosystems are characterized by dense animal communities that are primarily fueled by chemosynthetic bacterial productivity (1, 2). Many invertebrates adapt symbiotic lifestyles to facilitate their survival in extreme environments (3, 4). Episymbionts are multispecies consortia that occur as biofilms on host surfaces and have been identified on the hosts Alvinella pompejana (5), Rimicaris exoculata (6), Kiwaidae family species (7), and *Laminatubus* n. sp., among others (3, 8, 9). The episymbionts can supply hosts with organic carbon and other essential nutrients while also protecting the host from toxic compounds by degrading them (10).

Although the episymbiont communities are directedly exposed to environments, their species compositions significantly differ from those in adjacent nonhost environments (11). Further, the distribution of episymbionts on hosts is uneven because populations are enriched in specific parts of invertebrates (8). For example, episymbionts are selectively located on the dorsal epithelium of *Alvinella pompejana* (12, 13) and in the cephalothoracic cavity of *Rimicaris exoculata* (14–16). Further, considerable partner specificity between episymbionts and their hosts has been observed in *Niphargus* species (17) and Zoothamnium niveum (18), among others. In addition, hosts can exert control over their symbionts by modulating their social interactions or by direct immune control (19, 20). Thus, episymbiont communities are shaped by complex interactions of hosts, in addition to interactions among bacterial populations (21) that may further stabilize their coevolutionary patterns (22).

Microbe-microbe interactions, including cooperative, competitive, and predatory interactions have been suggested as critical for ensuring community specificity, particularly in open systems (23, 24). Among these interaction types, cooperation plays a particularly significant role in maintaining complex community structures (25), improving stress resistance (26), and increasing community productivity (27). The Black Queen Hypothesis states that bacterial populations unavoidably leak public goods (e.g., nutrients or metabolites) that are then available to the entire community and could lead to metabolite dependencies and adaptive gene loss and promote the coexistence of diverse bacterial taxa (25, 28). For example, dependency has been suggested to increase the coexistence of species via waste-product exploitation (29). Furthermore, these interdependent cooperative interactions provide considerable selective advantages to bacterial species (26, 30, 31). In episymbiotic communities, dominant chemosynthetic bacteria are primary producers and are more likely to exude public goods, thereby promoting metabolic interactions. However, little is known about the process of leaking compounds and the regulatory mechanisms of interactions, primarily due to difficulties in culturing episymbionts (32, 33).

Next-generation sequencing has greatly facilitated our understanding of uncultured microorganisms. However, metagenomics analyses can only provide information about metabolic potential, while other techniques, including imaging, physiological experiments, and transcriptomic analyses are needed to assess the actual activities of microorganisms in environments. For example, their biochemical composition and molecular characteristics can be directly observed using confocal Raman microscopy. Combined genome information of taxonomic groups with high-resolution imaging and characterization of their metabolites can shed light on how episymbionts interact with each other.

Shinkaia crosnieri (34) is a dominant animal species within hydrothermal vents and cold seeps along and near the Okinawa trough in the Northwest Pacific Ocean (35), and this is at least partially due to their close associations with episymbiont microorganisms on their seta (35, 36). 16S rRNA amplicon analysis and metatranscriptomic analyses have revealed that the episymbionts of *S. crosnieri* are dominated by bacteria in the orders *Thiotrichales*, *Campylobacterales*, and *Methylococcales*. The symbionts can supply their hosts with carbon through chemosynthetic carbon fixation using methane and sulfide as electron donors (36–42). However, natural stable carbon isotopic compositions of *S. crosnieri* epibionts differ extensively among habitats, with the mean delta13C‰ values of vent individuals ranging from −26.3 to −37.0 (41) and up to −54.1 within seep individuals. These observations suggest a higher abundance of potential epibiotic methanotrophs and a greater contribution of methanotrophic activity to epibiotic primary production of *S. crosnieri* populations in cold seeps. However, the composition of bacterial communities and their contributions to symbiont environment functioning are unknown in cold seep populations. Episymbiotic compositions are environmentally dependent (43, 44), and thus, samples from cold seeps are necessary to more thoroughly contextualize the episymbionts of *S. crosnieri*.

Here, metagenomic and metatranscriptomic analyses were conducted using five *S. crosnieri* individuals collected from a cold seep in the South China Sea to characterize the taxonomic composition and functions of their symbionts. Genome binning and reconstruction into metagenome-assembled-genomes (MAGs) were then conducted to identify the metabolic processes conducted by each symbiont and to hypothesize possible interactions among episymbionts. Interactions were subsequently evaluated using high-resolution imaging analysis, including fluorescent *in situ* hybridization (FISH) and confocal Raman microscopy. The potential roles of the interactions in the environmental adaptations of the symbionts were assessed, with particular attention paid to how symbionts survive in the oxic-hypoxic environmental shifts characteristic of cold seeps.

## RESULTS AND DISCUSSION

**FIG 1:**
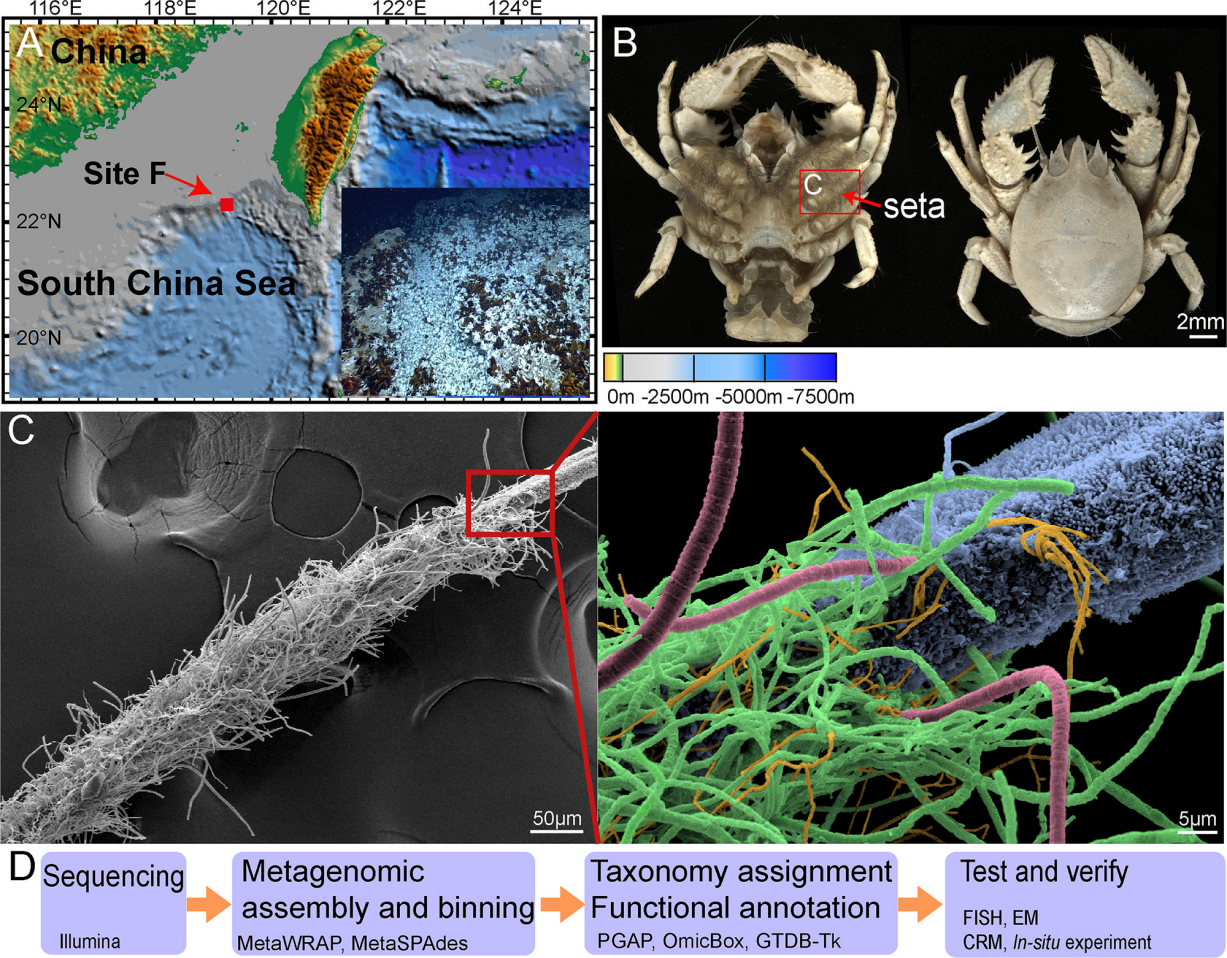
*Shinkaia crosnieri* seta symbiont sampling information. (A) The geographic location of the cold seep site studied here (i.e., the F site) and image of the *S. crosnieri* community adjacent to a methane seepage taken by the ROV *Faxian*. (B) Ventral and dorsal view of *S. crosnieri*, with seta on the appendages highlighted. (C) Scanning electron microscopy (SEM) image showing the distribution of key episymbiont taxa on the seta based on FISH analyses. Coloring of taxa is as follows: blue, *Methylococcales*; red, *Thiotrichales*; yellow, *Campylobacteriales*; green: *Methylococcales* or *Thiotrichales*. (D) Work flow for investigations of this study.

Metagenomic data for the *Shinkaia crosnieri* lobster episymbiont communities from the cold seep F site in the South China Sea were used here to reconstruct bacterial genomes. Further, these data were used to explore potential metabolic interactions within the microbial communities, in addition to the ecological effects of these interactions (Fig. 1).

### Taxonomic composition of *S. crosnieri* episymbiotic communities.

**FIG 2:**
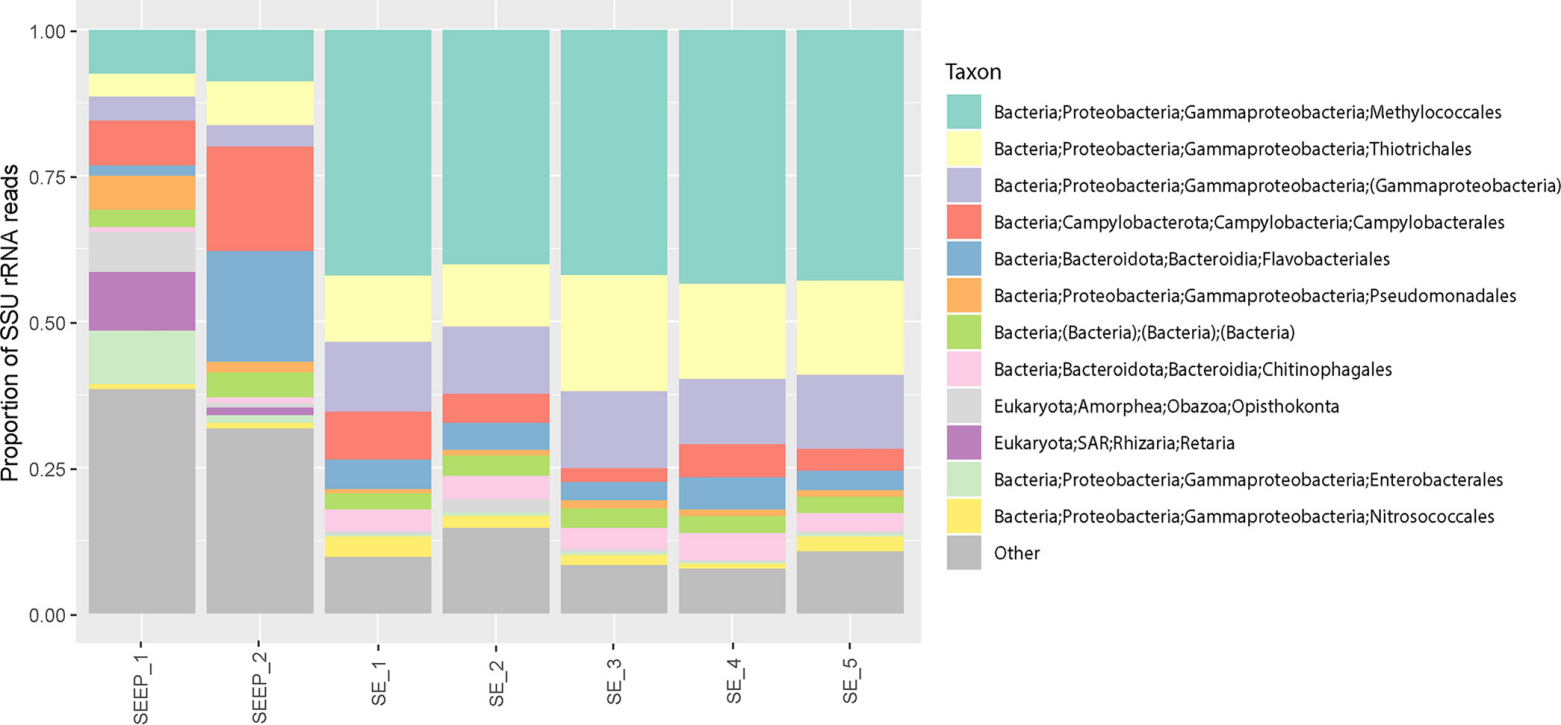
Comparison of order-level 16S rRNA gene phylotype relative abundances among *S. crosnieri* episymbionts and environmental microbial populations within lobster habitats. SEEP_1 and 2, environmental seep samples; SE_1 to 5, seta samples. Quantification was conducted with reconstructed SSU sequences from metagenomic sequencing data sets using phyloFlash.

The taxonomic compositions of the episymbiont communities were initially characterized with 101,880 small subunit (SSU) sequences using phyloFlash. Briefly, *Methylococcales* (42.3%) were the most abundant order, followed by *Thiotrichales* (14.8%), *Campylobacteriales* (4.9%), *Flavobacteriales* (4.4%), *Chitinophagales* (3.8%), and *Nitrosococcales* (2.2%) (Fig. 2). In comparison to an environmental water sample collected near the lobster’s habitat, episymbiont community structures were significantly different from environmental community structures, where the relative abundances of *Methylococcales* were much lower, and those of the *Campylobacteria* were much higher (see Fig. S1 in the supplemental material). These results suggest possible regulation in community structuring rather than episymbionts representing environmental population subsets (8, 9). The relative abundances of sulfur-oxidizing bacteria (SOB) in the vent samples range from 60% to 90% (45–47), which is much higher than that of episymbionts of *S. crosnieri* in our samples. In contrast, methane-oxidizing bacteria (MOB) relative abundances are much lower in the vent samples (8% to 30%) (41, 48), which possibly reflects the geochemical differences between the two habitats. Higher methane (1.5 mM) and lower sulfide (<1 mM) were observed in the lobster environment at the F site (49) than at the vents (50), suggesting the importance of the geochemical environment in shaping episymbiont community (43, 51).

10.1128/msystems.00320-22.1 FIG S1 Principal-coordinate analysis ordination of *S. crosnieri* episymbiont and environmental microbial community compositions based on 16S rRNA gene distributions. SE_1 to 5, seta samples; SEEP_1 to 3, environmental seep samples. The episymbionts and the seawater communities were separated well by PC1. Download FIG S1, TIF file, 0.6 MB. Copyright © 2022 Xu et al. 2022 Xu et al. https://creativecommons.org/licenses/by/4.0/ This content is distributed under the terms of the Creative Commons Attribution 4.0 International license.

The morphological features and spatial distributions of predominant populations were evaluated with FISH (Fig. 1E, Fig. S2). Samples were collected from the same individuals as described above, and sample characteristics were consistent among samples. Rod-shaped *Methylococcales* were attached on the seta surfaces or via the formation of filamentous aggregations with diameters between 0.8 and 1.5 μm. Both *Thiotrichales* and *Campylobacteriales* formed filament-like aggregations but differed in diameter with 2 to 3 μm and 0.4 to 0.6 μm diameters, respectively. *Flavobacteriales* were observed as small, rod-shaped cells attached to other filamentous bacteria, indicating that they may utilize the organic carbon generated by other chemosynthetic bacteria. These associations are consistent with observations in biofilms growing on the surfaces of a black smoker chimney in the Loki’s Castle vent field (52). Episymbiont biofilms tended to develop overlapping colonies with other bacteria that occur in close proximity that would thereby facilitate metabolite sharing among symbionts.

10.1128/msystems.00320-22.2 FIG S2 Confocal fluorescence microscope images of episymbionts on seta. (A) Episymbionts stained with DAPI. At least five different bacterial morphotypes were observed in the episymbiotic community. (B) *Methylococcales* symbiont hybridization with the Methy2 probe showing rod-shaped cells attached to the seta surfaces. The *Methylococcales* tended to be distributed at the tip of the seta where filamentous bacteria were rare. This group of bacteria included the type I methanotrophs, as evinced by the presence of intracellular stacked membranes in TEM images (Fig. 4). (C) *Methylococcales* episymbionts labeled with the Methy3 probe, showing square cells of approximately 1-μm diameter that formed filament-like aggregations. The *Methylococcales* episymbionts also included methanotrophs with intracellular stacked membranes. Many cells in the group were observed during the division stage, suggesting a high level of metabolic activity. (D and E) *Thiotrichales* episymbionts that hybridized with the Cocle (C) and Thrix (D) probes. The two groups of bacteria were characterized by discoid cells that formed filament-like aggregations but differed in diameter sizes of 0.8 to 1.2 μm and 2 to 3 μm, respectively. (F) *Flavobacteriales* episymbionts hybridized with the Flavo probe representing heterotrophs attached to the surface of other bacteria. (G) *Campylobacteriales* episymbionts hybridized with the EPSY549 probe, showing long rod-shaped cells that formed filament-like aggregations with a diameter of 0.4 to 0.6 μm. Download FIG S2, JPG file, 1.9 MB. Copyright © 2022 Xu et al. 2022 Xu et al. https://creativecommons.org/licenses/by/4.0/ This content is distributed under the terms of the Creative Commons Attribution 4.0 International license.

### MAG construction and metabolic potential of *S. crosnieri* episymbionts.

A total of 97 MAGs were recovered by genome binning, with 81 exhibiting estimated completeness of >70% and contamination of <20% (Table S1). The raw-sequencing read mapping rates ranged between 64% and 76% for each sample (Table 1), suggesting good MAG representation among the samples. MAG sizes ranged from 1.39 Mbp to 9.17 Mbp. Only genomes with relative abundances of >1% were used for further analysis to focus on populations that most contributed to ecosystem functioning, comprising a total of 28 MAGs. Previous studies have shown that contamination of *Campylobacterota* MAGs can be extensive (53). Consequently, we relaxed the binning criteria to generate more *Campylobacterota* MAGs. Despite that, 20 of 28 MAGs are of high quality with completeness of >85% and contamination of <5%; assessments of gene absence should still be treated tentatively. To generate robust conclusions, MAGs from each order were considered a single group to provide greater confidence in the metabolic predictions. Additionally, the identities of target genes were evaluated with phylogenetic analysis to confirm that they belonged to the expected taxa. Moreover, the phylogenetic identities of genes adjacent to the target genes were evaluated to ensure that the contig containing the target genes was not an artifact.

**TABLE 1:** Metagenomic data set statistics

Sample ID	Total no. of spots	Total bases (Gb)	GC percentage	PE reads	MAG mapping rate (%)
SE_1	44,290,218	13.3	38.89	100,212,568	70.58
SE_2	34,357,365	10.3	40.03	61,833,808	64.11
SE_3	33,804,887	10.1	38.96	84,768,460	75.70
SE_4	42,166,925	12.6	38.22	107,130,404	75.12
SE_5	48,032,565	14.4	39.5	119,245,808	72.36

10.1128/msystems.00320-22.8 TABLE S1 MAG characterizations Table S1, XLSX file, 0.02 MB. Copyright © 2022 Xu et al. 2022 Xu et al. https://creativecommons.org/licenses/by/4.0/ This content is distributed under the terms of the Creative Commons Attribution 4.0 International license.

**FIG 3:**
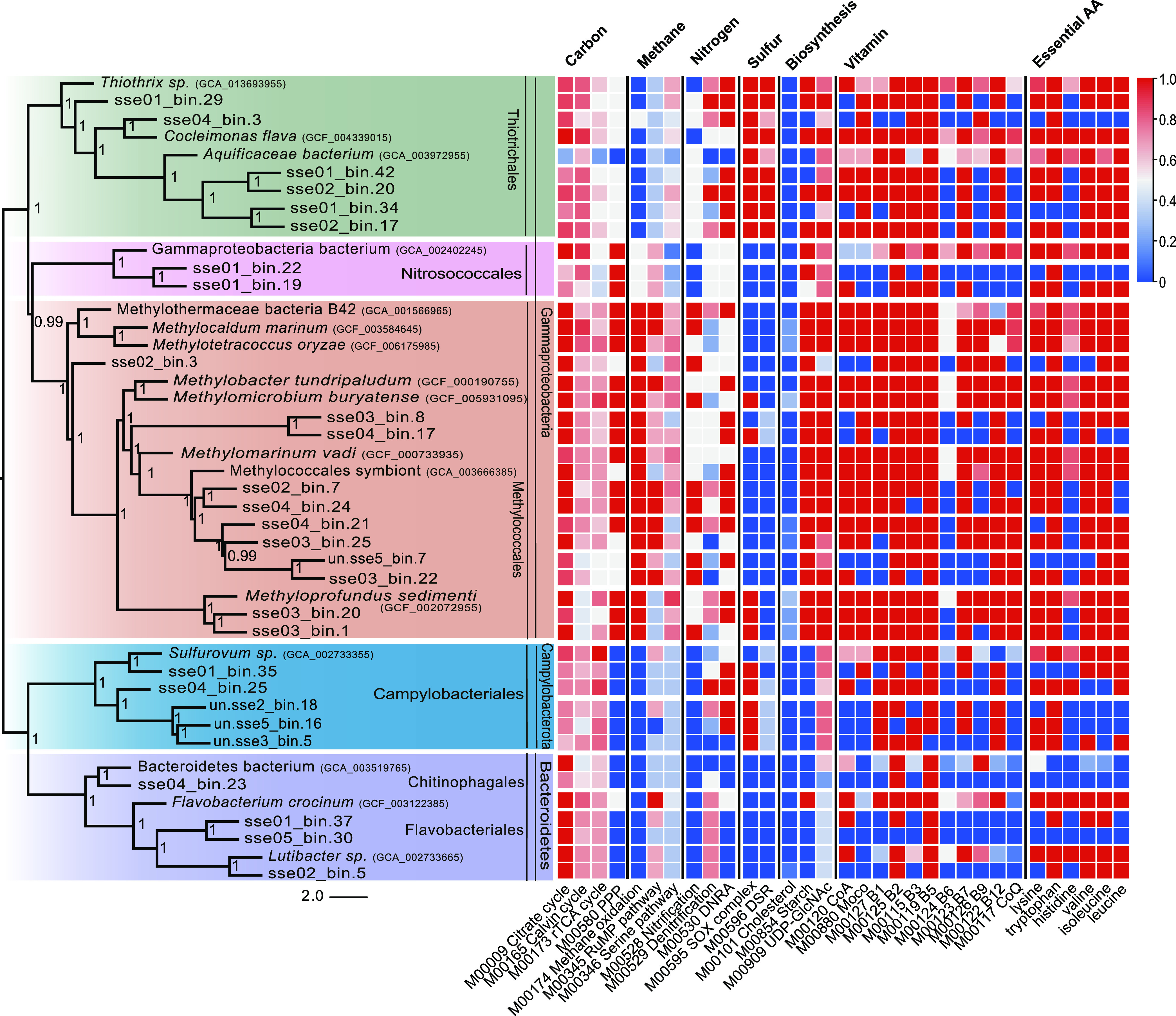
Phylogenomic assignment, relative abundance, and metabolic potential of the dominant MAGs of the setae along with reference genomes. Phylogeny of 28 high-quality MAGs recovered from the *S. crosnieri* setae. The colors of the tree represent order-level taxonomic groups for MAGs. Heat map colors represent completeness of KEGG modules.

Phylogenomic analysis indicated that the 28 MAGs clustered into five monophyletic clades in the orders *Thiotrichales*, *Nitrosococcales*, *Methylococcales*, *Chitinophagales*, and *Flavobacteriales* (Fig. 3), which represented all of the dominant taxa recovered in phyloFlash 16S rRNA gene-based analysis. The Genome Taxonomy Database Toolkit (GTDB-Tk)-based taxonomic assessments indicated that many MAGs were divergent from known reference genomes, suggesting that they may represent unknown genera and even unknown families.

The metabolic potentials of the dominant taxa are represented based on KEGG module completeness visualizations in Fig. 3. One-carbon metabolic pathways, including methane oxidation, methanol oxidation, and the ribulose monophosphate (RuMP) pathway were the most notable functional categories encoded by the *Methylococcales* and *Nitrosococcales* MAGs. Methane oxidation was particularly prominent in the *Methylococcales* MAGs. The *Thiotrichales* and *Campylobacteriales* can fix carbon dioxide via the reverse tricarboxylic acid (rTCA) and Calvin-Benson-Bassham (CBB) pathways, respectively, using electrons obtained by the oxidation of reduced sulfur. The *Flavobacteriales* and *Chitinophagales* MAGs both represented denitrifying heterotrophic bacteria that may metabolize a substantial fraction of organic carbon produced by primary production or by the hosts.

MAGs belonging to the same clade usually exhibited similar metabolic potentials. Thus, each order can be reasonably regarded as a functional group, and their metabolic functions could be generally compared. The abundances of significantly different metabolic modules among different orders were tested using nonparametric analysis of variance (ANOVA) (Fig. S3), with those identified as significant, including modules related to electron donor/acceptors, carbon fixation pathways, amino acid biosynthesis, and vitamin biosynthesis. High levels of expression (top 200) for key genes in these pathways indicated that the pathways were active *in situ* (summarized in Fig. S4).

10.1128/msystems.00320-22.3 FIG S3 Heat map showing pathway module completeness. Only significantly different pathways among five orders (based on ANOVA analysis) are shown. The color scale indicates module completeness. The functional modules for fundamental biological processes such as ribosomal structures, glycolysis, and nucleotide metabolism were complete in most MAGs, confirming a high data set quality. The completeness values of MAGs in the same order were generally similar, with the exception of *Nitrosococcales* and *Campylobacteriales* MAGs that were more variable. Thus, each order can be considered a functional group, and their metabolic functions were comparable. Modules for amino acid biosynthesis and degradation, coenzyme metabolism, and electron transport chain were variable. For example, different cytochrome *c* oxidases were carried by different orders. Download FIG S3, TIF file, 1.0 MB. Copyright © 2022 Xu et al. 2022 Xu et al. https://creativecommons.org/licenses/by/4.0/ This content is distributed under the terms of the Creative Commons Attribution 4.0 International license.

10.1128/msystems.00320-22.4 FIG S4 Overview of the central metabolic pathways of episymbionts. The gene expression levels of each KEGG ortholog were calculated based on the FPKM metric for corresponding MAGs of each order. The expression values were ranked to show relative contributions, as indicated by the color legend. Genes related to methane oxidization were most highly expressed by the *Methylococcales*, followed by genes related to formaldehyde assimilation and genes related to starch biosynthesis. Key genes of the Calvin-Benson-Bassham cycle (*rbcLS*) were highly expressed by *Thiotrichales*, while key reverse TCA cycle (*ACLY*) and gluconeogenesis (*kor*, *por*) genes were highly expressed by *Campylobacteriales*. Among sulfur metabolism pathways, genes for sulfide oxidation such as *sqr* and *fcc* were highly expressed by most episymbionts. High expression levels of genes in the reverse Dsr pathway were only observed for *Thiotrichales* populations, while SOX complex gene expression was observed for both *Thiotrichales* and *Campylobacteriales*. Relatively low expression of genes in the SOX complex was also observed for *Methylococcales* episymbionts, suggesting their potential for thiosulfate oxidation, as discussed in the text. Regarding nitrogen metabolism, possible evidence at the expression level was observed that some intermediates are shared, leading to complete pathway activities. Ammonia could be oxidized by *Methylococcales* using enzymes encoded by *pmoA* and *hao* or oxidized by *Campylobacteriales* via NrfA. The expression of nitrogen fixation genes was not observed in this study, consistent with the metagenomic analyses. In addition, the expression of different amino acid biosynthesis genes was considerably variable. Further, glutamine and aspartate biosynthesis genes were highly expressed in the majority of episymbionts, while alanine biosynthesis genes were only highly expressed by the *Thiotrichales*. Acetyl-CoA synthetase genes were highly expressed by the *Methylococcales* and *Thiotrichales*, suggesting that micromolecular hydrocarbons such as acetate could serve as supplementary energy sources for these deep-sea chemosynthetic microbial communities. Download FIG S4, TIF file, 0.2 MB. Copyright © 2022 Xu et al. 2022 Xu et al. https://creativecommons.org/licenses/by/4.0/ This content is distributed under the terms of the Creative Commons Attribution 4.0 International license.

### Carbon fixation.

Both the *Methylococcales* and *Nitrosococcales* MAGs encoded a complete set of genes for the RuMP pathway, enabling the fixation of carbon through formaldehyde assimilation (Fig. 3, carbon and methane). However, the *Nitrosococcales* did not carry particle methane monooxygenase (*pmoABC*) or soluble methane monooxygenase (*mmoXYZ*) genes and thus might require the acquisition of methanol, formaldehyde, or other methyl compounds from surrounding environments. These results suggest that the *Nitrosococcales* may partially rely on methanol leaked from *Methylococcales* cells, as previously observed in a eutrophic lake water system (54). Similarly, type I gammaproteobacterial *Methylococcaceae* dominated oxic and suboxic bottom waters and surface sediments among five lakes in central Switzerland, exhibiting strong correlations with the abundances of putatively methylotrophic *Methylophilaceae* (55). Isotopic labeling experiments have previously shown that methanol is a small, weakly polar molecule that can diffuse across cell membranes (54). In addition, *pmoABC* were some of the most highly expressed genes in the *Methylococcales*, suggesting rapid methane oxidation to methanol that could serve as a public good that would benefit *Nitrosococcales*.

Genes involved in the serine pathway were identified in the *Methylococcales* and *Thiotrichales* MAGs. However, none of the MAGs encoded the complete pathway, suggesting that the serine pathway was nonfunctional, as previously suggested (42). *Thiotrichales* carried complete gene sets for Calvin-Benson-Bassham (CBB) cycles, while most *Campylobacteriales* could fix carbon through the rTCA cycle with ATP-citrate lyase. Thus, the two orders can fix organic carbon from carbon dioxide. The *Bacteroidetes* MAGs appeared to be heterotrophic. Regarding the other heterotrophs, the *Flavobacteriales* MAGs carried genes for amino acid degradation, while the *Chitinophagales* MAGs encoded chitinase that could enable chitin degradation, which is the main component of lobster crusts and setae. Corresponding genes of those indicated above were also identified in the metatranscriptomic data sets with relatively high abundance (Table S2).

10.1128/msystems.00320-22.9 TABLE S2 Metatranscriptomic abundances of key genes within metabolic pathways among MAGs Table S2, XLSX file, 0.1 MB. Copyright © 2022 Xu et al. 2022 Xu et al. https://creativecommons.org/licenses/by/4.0/ This content is distributed under the terms of the Creative Commons Attribution 4.0 International license.

### Sulfur metabolism.

Complete sulfur metabolism-related modules were identified in the episymbiont MAGs (Fig. 3, sulfur). In particular, the *Thiotrichales* MAGs encoded two periplasmic enzymes, sulfide:quinone oxidoreductase (*sqr*) and flavocytochrome *c* (*fccB*), in addition to a partial SOX complex (*soxCD*-deficient) that could oxidize sulfide and thiosulfate to elemental sulfur. The MAGs also carried genes necessary for the reverse dissimilatory sulfite reductase (rDSR) pathway that would enable the oxidation of intermediate sulfur forms in *soxCD*-deficient bacteria. Most *Campylobacteriales* carried *sqr*, *fccB*, and complete SOX complexes.

Carbon fixation modules were absent in the *Methylococcales* MAGs, although sulfur-oxidizing modules were present, including *sqr* and *fccB*, in addition to the cytoplasmic enzyme sulfur dioxygenase (*sdo*). High concentrations of sulfide inhibit respiratory pathways and methane oxidation (56). Consequently, these sulfide-oxidizing enzymes could be used for detoxification rather than catabolism (57, 58). Further, sulfide-oxidizing enzymes can also provide a supplementary electron pool (59). Two *Methyloprofundus* MAGs and a *Methylococcales* MAG that could not be assigned to a genus encoded SOX complexes for thiosulfate oxidation (Fig. 3). These genes were colocalized, consistent with their presence in other *Methylococcales* genomes (Fig. S5), while also exhibiting close identity to genes within other methylotrophic bacterial genomes (60). Consequently, the SOX complexes in the methylotrophic MAGs are not likely data analysis artifacts. All the aforementioned genes were actively transcribed, based on transcriptomic data. Consequently, participation of *Methylococcales* in sulfur cycling within these symbiont communities should be considered (61).

10.1128/msystems.00320-22.5 FIG S5 (A and B) Schematic illustration (A) of the loci comprising *soxB* genes in a methanotrophic MAG (sse04_bin.17) and sequence alignments (B) of *soxB* with references from the GenBank database. The red arrows indicate genes taxonomically annotated as *Methylococcaceae*. The green arrows indicate genes not most closely related to methanotrophs. BLAST comparisons indicated that the *soxB* genes were most similar to genes encoded by Methylobacter marinus, Methylobacter luteus, and a *Methyloglobulus* sp. within the *Methylococcaceae*. Overall, these results indicate that *soxB* was encoded by *Methylococcales* episymbionts rather than representing a mis-binned analytical artefact. Download FIG S5, TIF file, 0.6 MB. Copyright © 2022 Xu et al. 2022 Xu et al. https://creativecommons.org/licenses/by/4.0/ This content is distributed under the terms of the Creative Commons Attribution 4.0 International license.

These hypothesized interactions may also connect SOB with other bacteria by the sharing of stable sulfur intermediates (Fig. 3, sulfur). All symbiont MAGs encoded either *sqr* or *fccB* genes that catalyze sulfide oxidation to sulfur. However, only *Thiotrichales* and *Campylobacteriales* populations exhibited the ability to fix carbon through sulfur-compound oxidation. Thus, SOB may utilize sulfur intermediates produced by other bacteria. Sulfur-sulfur bond signals were observed in the Raman confocal images of *Methylococcales* cells, confirming that elemental sulfur or persulfides are synthesized in MOB. In addition, complete genetic pathways related to sulfur relay systems and glutathione *S*-transferases were highly expressed in the *Methylococcales* and *Thiotrichales*, suggesting the transport of elemental sulfur or persulfides across membranes for enzymatic reactions (62) between symbionts. Sulfur granule protein (*sgp*) genes were encoded by the *Thiotrichales* MAGs, suggesting that they can stably store elemental sulfur inside cells. Thus, we hypothesize that the *Thiotrichales* can further utilize sulfur derived from other cells, and especially the MOB, as suggested by the close proximity of these cells in FISH.

### Nitrogen metabolism.

The primary nitrogen metabolism modules identified in the episymbiont MAGs are shown in Fig. 3 (nitrogen). Nitrate-ammonifying (DNRA) modules were only observed and transcribed in chemosynthetic bacterial MAGs, although denitrification-related genes were observed in the MAGs of all dominant orders of the symbiotic communities. The *Methylococcales* MAGs, with the exception of three MAGs in a subcluster, exhibited the capacity for partial denitrification, with the last enzyme (NosZ) required to convert N_2_O to N_2_ absent in the former genomes. Nitrate reductases were also encoded by the MAGs and highly expressed (Table S2) in most chemosynthetic symbionts but were absent in the *Bacteroidetes* MAGs. However, the chemosynthetic orders utilized different enzymatic complex types, with the *Methylococcales* encoding Nar-type enzymes that couple nitrate respiration to proton translocation across the membrane. MAGs of the other two orders encoded Nap-type enzymes that do not directly generate proton motive forces (63). The *Bacteroidetes* MAGs did not encode nitrate reductases and thus might utilize nitrite sourced from the environment to complete the denitrification pathway.

Most *Methylococcales* symbionts exhibited evidence for nitrification, based on mostly complete corresponding modules. Ammonia monooxygenase (AMO) and methane monooxygenase (MMO) are homologous enzymes, such that ammonia can serve as an alternative substrate for methane in *pmoABC* (64). A previous study demonstrated that methane consumption by type I methanotrophic oxidation bacteria was enhanced via ammonium supplementation to tidal flat sediments of the Yangtze River estuary (65). A high concentration of ammonia was observed in the waters that the invertebrate communities inhabited. Thus, the potential contribution of ammonia as a substrate for cold-seep ecosystem productivity may be underestimated.

**FIG 4:**
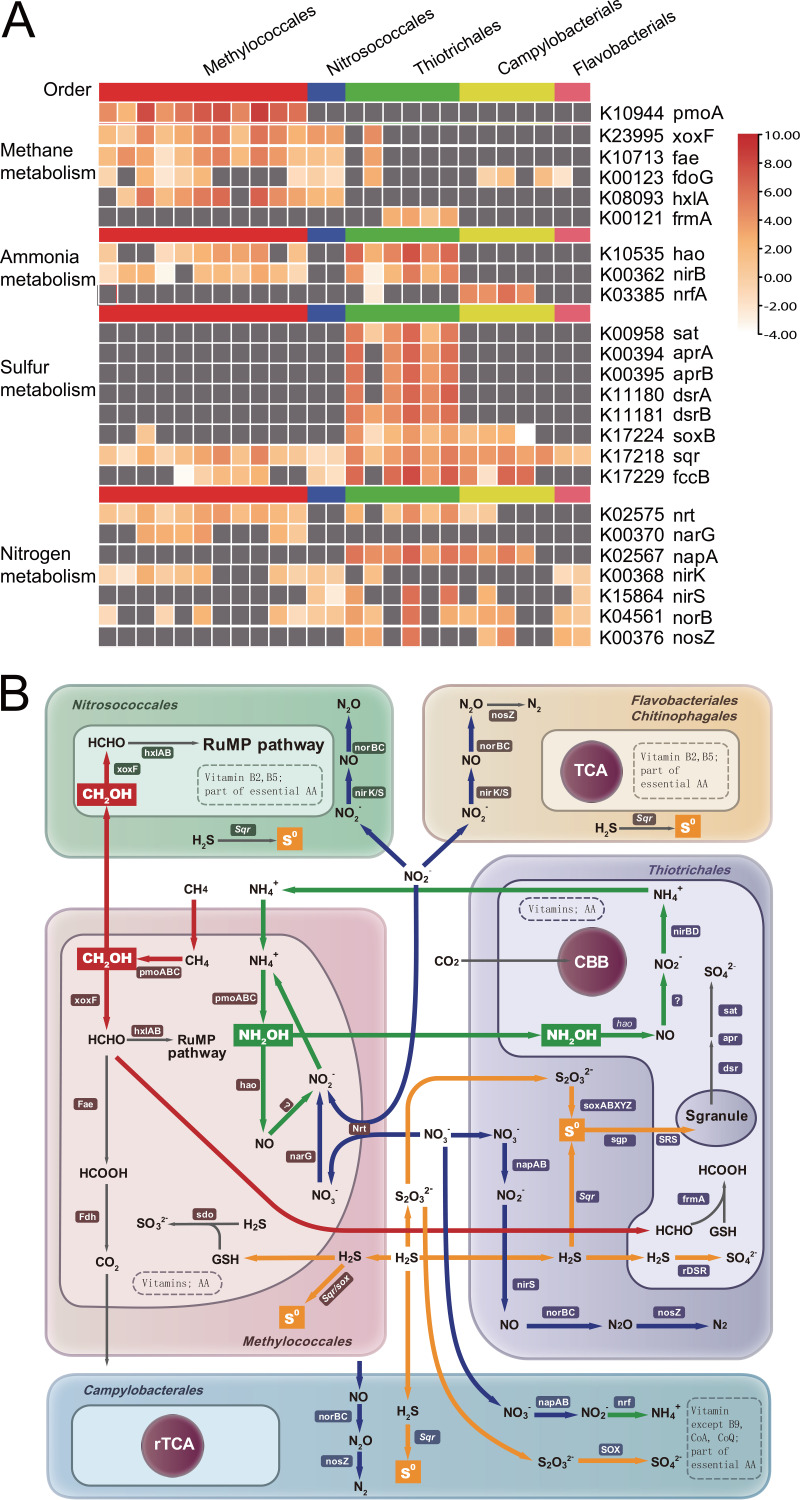
Conceptual schemes showing proposed interactions among representative episymbiont orders inhabiting the *S. crosnieri* setae. (A) Relative expression of critical genes normalized to RPKM values. Gray indicates a gene absent in the transcriptomic data set. (B) Major metabolic pathways predicted to be utilized by the dominant orders in addition to inferred shared goods among symbionts. Individual pathways are indicated with the same color. Detailed descriptions of pathways are provided in Table S2. SRS, sulfur relay system; GSH, glutathione; AA, amino acid.

**FIG 5:**
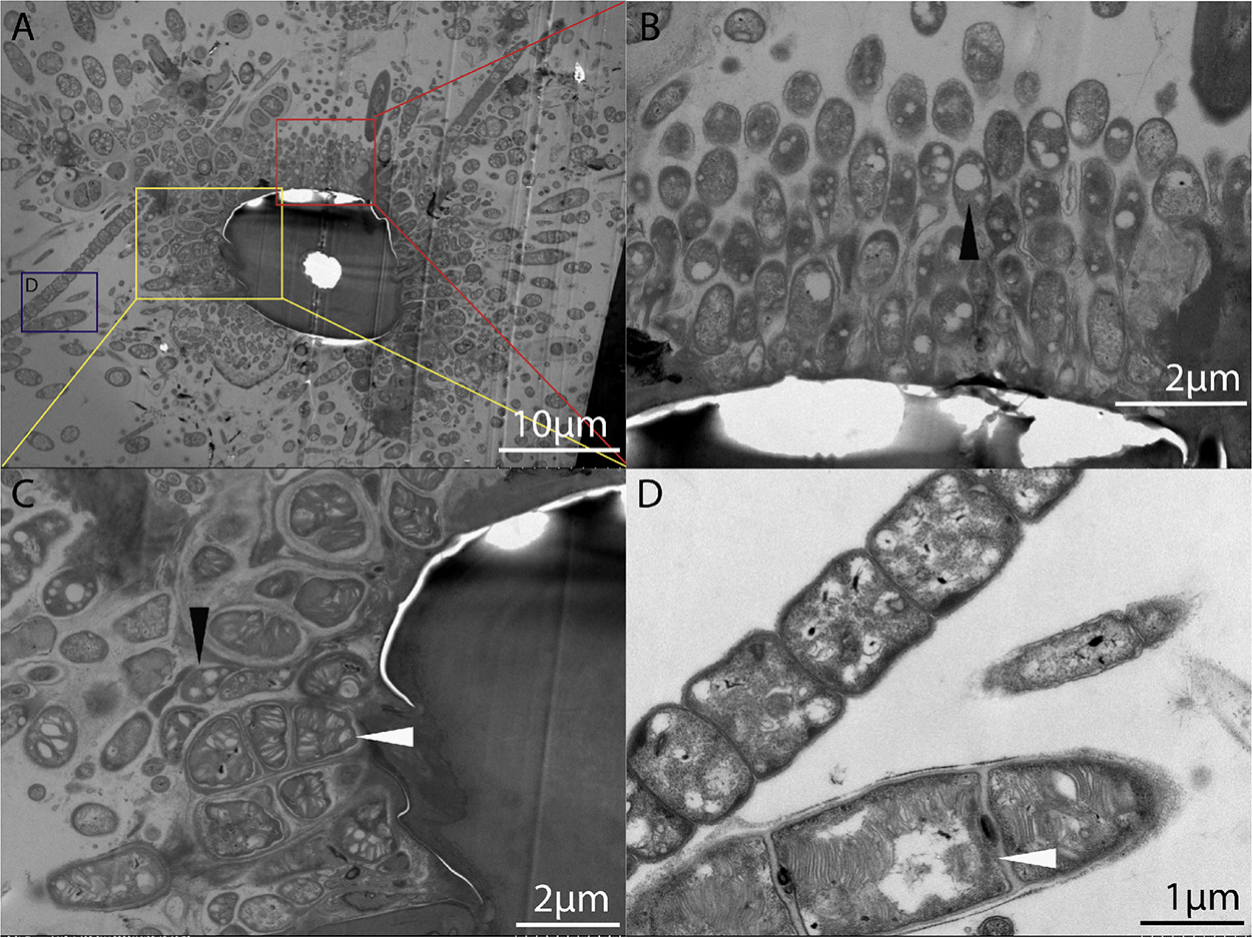
Transmission electron micrographs of *S. crosnieri* setae episymbionts. (A) Overview of the setae and associated bacterial cells. (B to D) Observed morphotypes. White arrow, methanotrophs with intracellular stacked membranes that are typical of type I methanotrophs. Black arrow, *Thiotrichales* cells harboring intracellular sulfur globules.

The abundant MMO enzymes carried by *Methylococcales* could be used to transfer ammonia to hydroxylamine via monooxygenation, since the latter is toxic and mutagenic (61, 66). Furthermore, hydroxylamine is an intermediate that can be released by aerobic ammonium-oxidizing bacteria and used by other community members (67, 68). Hydroxylamine can transiently accumulate in ammonia-oxidizing bacterial planktonic populations or mixed cultures and is considered a key nitrogen metabolite in microbial interactions within microbial communities and engineered systems (69–71). Accordingly, the presence and high expression levels (Fig. 4A) of hydroxylamine dehydrogenase (*hao*) by *Thiotrichales* populations may be associated with hydroxylamine detoxification. Thus, the *Thiotrichales* may contribute to hydroxylamine detoxification in the episymbiont community. The proportion of *Thiotrichales* that carried *hao* was much higher in our data set than in all *Thiotrichales* genomes in the NCBI database (21 of 22 here versus 5 of 1,756 in the NCBI genome database). The enrichment of *hao* in symbiotic *Thiotrichales* suggests a possible beneficial function of the enzyme in improving the overall activity of the episymbiotic community through hydroxylamine elimination. Similarly, the *Thiotrichales* MAGs encoded glutathione-dependent formaldehyde dehydrogenases (*frmA*) (Table S2) that can function in formaldehyde detoxification (72). Thus, the *Thiotrichales* and *Methylococcales* are the symbiotic community’s two most abundant taxa and may have established metabolic interactions that benefit both populations. The distributional overlap observed for these two taxa was also observed via transmission electron microscopy (TEM), further suggesting potential interactions between the two (Fig. 5).

### Biosynthesis of essential amino acids and coenzymes.

The metagenomic data were used to evaluate the ability of different taxa to synthesize essential biomolecules (Fig. 3, biosynthesis, vitamin, essential amino acid). The episymbionts exhibited the potential to provide most nutrients to their hosts. In return, the invertebrate hosts could supply vitamin B_6_ to episymbionts, since it is not synthesized by any of them (73). Chemotrophic episymbionts synthesized broader complements of essential molecules, while the heterotrophic episymbiont MAGs lacked genes for the biosynthesis of most amino acids and coenzymes. Thus, the heterotrophic episymbionts may have established close metabolic associations with their chemosynthetic symbionts. For example, the *Flavobacteriales* and *Chitinophagales* need to externally obtain coenzymes such as coenzyme A, vitamin B_1_, and amino acids, including lysine and histidine. *Methylococcales* exhibited greater versatility in nutritional synthesis, with the capacity to generate most vitamins and essential amino acids. In addition, only the *Methylococcales* encoded the capacity to synthesize the cholesterol precursor squalene. The *Thiotrichales* MAGs did not encode genes for vitamin B_6_, vitamin B_9_, coenzyme Q, and histidine biosynthesis. In addition, the *Campylobacteriales* appear to greatly rely on the host and other bacteria in the community due to their lack of encoded capacity to biosynthesize coenzyme A, molybdenum cofactor, vitamin B_6_, vitamin B_9_, coenzyme Q, histidine, valine, and isoleucine. Taken together, these results suggest possible nutritional cooperative interactions within the episymbiotic community, as proposed by the Black Queen Hypothesis (28).

The nutrient contributions to the host are also likely complementary. For example, the essential amino acid histidine could only be provided by some *Campylobacteriales* and *Thiotrichales*, and the steroid precursor lanosterol could only be synthesized by the *Methylococcales*. The complex functionally complemented symbiotic community may be adaptive and selected by the host, although the regulatory mechanisms of episymbionts remain unclear (74).

### Potential metabolic interactions.

The symbionts in the communities analyzed here exhibited the ability to conduct nutrient cycle components, wherein intermediates could be further metabolized by other community members, thereby enabling the completion of whole pathways (Fig. 4). For example, *Nitrosococcales* may partially rely on methanol leaked from *Methylococcales* cells. SOB may also be connected with other bacteria by sharing stable sulfur intermediates. Further, the enrichment of *hao* and *frmA* in symbiotic *Thiotrichales* suggests a possible beneficial enzymatic function of detoxification. Moreover, the heterotrophic episymbiont MAGs lacked genes for the biosynthesis of most amino acids and coenzymes that could be synthesized by chemotrophic episymbionts and hosts, suggesting a metabolic reliance of the heterotrophs on the chemotrophs and hosts. The above-described results consequently provide a real-world example of the Black Queen Hypothesis (28) in a deep-sea chemosynthetic ecosystem (Fig. 4B). Specifically, C_1_ compounds, sulfur intermediates, and other nutrients are likely shared among community members. These public goods available to the entire community may lead to metabolite dependencies among populations. Thus, cooperative interactions could possibly lead to selective advantages of bacteria.

Chemoautotrophic populations exhibit unique metabolic characteristics. For example, organotrophic *Bacteroidetes* attach to and glide along lithoautotrophic *Sulfurovum* filament surfaces while also utilizing organic polymers produced by the *Sulfurovum* growing on the surface of a black smoker chimney in the Loki’s Castle vent field (52). Furthermore, the symbiont community of sponges may improve overall nitrogen utilization efficiency in the host through organic nitrogen cycling.

Most known chemosynthetic symbioses involve SOB, while MOB-based symbioses are far less common (4, 9, 75). In an MOB-dominated biofilm community, leaking methanol and formaldehyde from methanotrophs can support methylotrophs. Further, reduced sulfur produced by MOB can provide an energy substrate for SOB. In return, flourishing SOB can benefit the MOB via sulfide detoxification. These positive feedback interactions may enhance the cooperation of episymbiotic communities. However, insufficient information is available regarding the metabolic pathways of vent episymbiont communities. The communities at vents or shallow waters are primarily dominated by SOB (primarily within the *Gammaprotebacteria* and *Campylobacteria* divisions), suggesting divergent characteristics of metabolic interactions.

### Metabolic adaptations of *S. crosnieri* episymbionts toward fluctuating environments.

**FIG 6:**
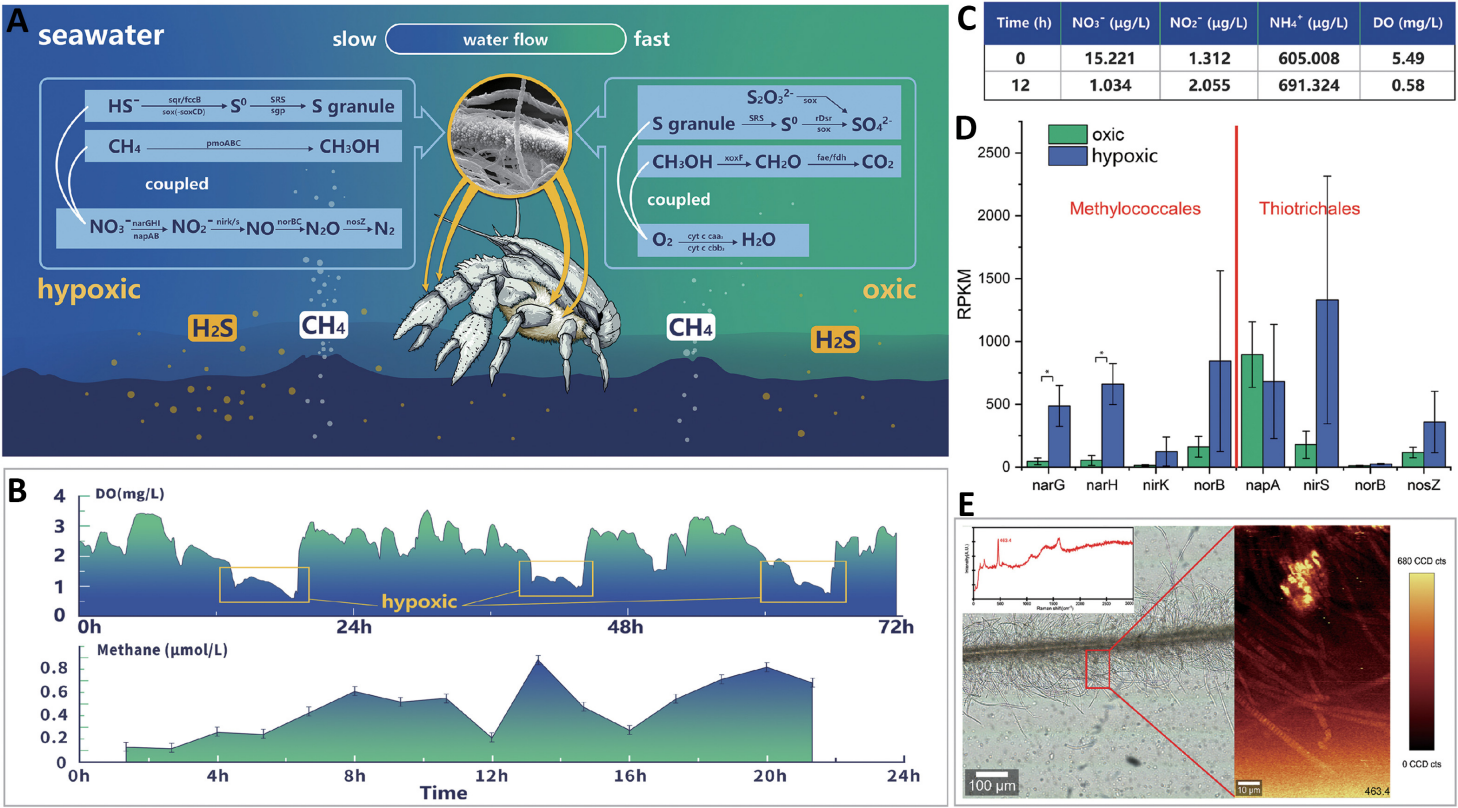
Metabolic strategies adopted by *S. crosnieri* episymbionts in oxic-hypoxic fluctuating environments. (A) Schematic of the predicted overall community strategy. During the hypoxic phase, the symbionts oxidize methane and sulfide to methanol and sulfur globules by coupling to nitrate respiration. The stable sulfur and methane intermediates can then be stored as inclusions or released to the environment and taken up by other symbionts. During the oxic phase, episymbionts can use oxygen to oxidize sulfur and methanol to conserve additional energy to support cellular growth. (B) Temporal measurements of DO and methane concentrations inside the *S. crosnieri* community. (C) Nutrient and dissolved oxygen concentrations of environmental seawaters. (D) Gene expression comparison among episymbionts under oxic and hypoxic conditions within *in situ* experiments. (E) Confocal Raman microscopy imaging showing elemental sulfur in the episymbiont communities.

Environmental conditions are unstable in cold seeps due to temporal variation of reduced compounds that are released from the bottom currents that could further shape regional chemical spatial distributions in the subseafloor (2, 76–78). Temporal dynamics of methane and oxygen (Fig. 6B) in the *S. crosnieri* assemblies were evaluated and confirmed the presence of oxic/microoxic transitions that periodically occur inside the community. Oxygen concentrations ranged from 0.6 to 3.5 mg/L, while methane concentrations varied between 0.2 and 1 μmol. Methane concentrations were negatively correlated with oxygen concentrations (45). However, the lobster symbionts require oxygen and reduced substrates such as methane to efficiently fix carbon, which may be simultaneously unavailable in their natural habitats. Thus, different strategies could be used by the chemosynthetic symbionts to solve this paradox and achieve more efficient energy conservation. For example, the episymbionts could conserve energy through the oxidation of diverse electron donors (sulfide, thiosulfate, sulfur, methane, ammonia, and hydrogen) coupled with oxygen or nitrate respiration (Table S2), thereby allowing the cells to utilize adaptations to both oxic and hypoxic environments (79). Sulfur globules were observed in our samples (Fig. 6E). Although methanol was not directly quantified in these samples, it is reasonable to speculate that methanol accumulation occurred in the MOBs since *pmoABC* was expressed at 20-fold higher levels than lanthanide-containing methanol dehydrogenases (*xoxF*) under hypoxic conditions (Fig. 4A). In addition, globin-like proteins containing heme domains that may sense O_2_ (80) were encoded by many of the symbiont MAGs and could facilitate their dynamic responses to fluctuating oxygen environments (Table S2).

A deep-sea *in situ* experiment was conducted to evaluate the responses of the symbionts to hypoxia (Fig. S6B). Elevated nitrate respiration was observed under low oxygen conditions. Specifically, nitrate concentrations decreased while nitrite and ammonia concentrations extensively increased in the hypoxia treatment (Fig. 6C). Meanwhile, all denitrification genes were significantly upregulated in the *Methylococcales* under hypoxic conditions (Fig. 6D). The response of *napAB* expression to oxygen levels has been observed in Alcaligenes eutrophus strain H16 (81). Although the expression of *napAB* genes carried by *Thiotrichales* MAGs was not significantly influenced by hypoxia, other genes involved in nitrate respiration were considerably upregulated. The transcriptomic data further supported the contribution of nitrate respiration to productivity in the hypoxic environments. Thus, nitrate could function as an alternative electron acceptor during the hypoxic phase.

10.1128/msystems.00320-22.6 FIG S6 Deep-sea *in situ* incubation device manipulated by the sampling ROV. (A) 1, cover; 2, barrel; 3, snap-fit; 4, trigger of the mixer; 5, handle; 6, decompression valve; 7, ball valve; 8, plastic bag. Put sample individuals into the barrel, close the cover, and then close the ball valve. When the trigger of the mixer is pushed, the solution stored in the plastic bag is released into the tank. (B) Photo of the *in situ* hypoxic incubation experiment at the cold seep F site captured by the ROV *Faxian*. A total of four *S. crosnieri* lobsters were reared in the incubation device. A DO sensor was maintained in the incubator throughout the experiment. Download FIG S6, JPG file, 0.8 MB. Copyright © 2022 Xu et al. 2022 Xu et al. https://creativecommons.org/licenses/by/4.0/ This content is distributed under the terms of the Creative Commons Attribution 4.0 International license.

Here, we propose a conceptual community interactive model in which symbionts drive carbon fixation and biogeochemical cycling in staggered states, with stable intermediates of reductive compounds acting as intermediates (Fig. 6A). During the hypoxic phase, the symbionts oxidize methane and sulfide to methanol and sulfur globules by coupling to nitrate respiration. The stable sulfur and methane intermediates could then be stored as inclusions (82) or released to the environment and taken up by other symbionts (54). During the oxic phase, episymbionts can use oxygen to oxidize sulfur and methanol to conserve additional energy to support cellular growth. Hence, the stable intermediates likely play essential roles in metabolic interactions and adaptations to fluctuating environments that commonly occur at vents and seeps. The cooperation of the episymbiotic communities could lead to greater adaptability to chemosynthetic ecosystems, thereby providing greater levels of energy supplies to invertebrate hosts.

### Conclusions.

Genome-guided transcriptomic analysis was used to investigate episymbiont community species compositions and their potential metabolic functions. These results highlight the potential interactions among episymbiont taxa via complementary encoded enzymes within some pathways. In many cases, individual symbionts could only complete part of a metabolic pathway, although the intermediates could be relayed to another population to achieve the completion of certain pathways. In particular, the *Thiotrichales* genomes carried *hao*, which may detoxify hydroxylamine that is a by-product of *Methylococcales*. Stable intermediate compounds of metabolism such as elemental sulfur and methanol may be shared among symbionts, thereby benefitting the overall productivity of the community. For example, methanol and sulfur derived from the *Methylococcales* could provide substrates for *Nitrosococcales* and SOBs. Such interactions could contribute to the survival of symbionts in the dynamic environments that are typical of cold seeps. Likewise, symbionts may switch their metabolic activities to adapt to oxic or hypoxic phases. Reductive compounds could be partially oxidized coupled with nitrate respiration under hypoxic conditions, while the stable intermediates could be completely oxidized by oxygen under subsequent oxic conditions. Overall, these results provide an example of the Black Queen Hypothesis in deep-sea cold seep ecosystems and highlight the need to study holobionts as whole communities to understand their adaptations to extreme environments.

## MATERIALS AND METHODS

### Sample collection and data generation.

*Shinkaia crosnieri* bacteria were collected in August 2018 from a cold seep (referred to as the F site) in the South China Sea (119°17′8.22″ E, 22°06′55.26″ N, depth 1,120 m) by the remotely operated vehicle (ROV) *Faxian*, which was on board the scientific research vessel *Kexue* (Fig. 1A). The samples were taken on board in around 45 min. Immediately after being taken onboard, the lobsters were put into precooled seawater and were transferred to the laboratory. The lobsters were thoroughly rinsed three times with sterile seawater. Then the plumose setae (Fig. 1B and C) on pereopods were dissected with sterilized scissors and then were flash-frozen in liquid nitrogen. All the laboratory processes were finished within 30 min. The setae of five lobsters were divided into three components. One portion was flash-frozen in liquid nitrogen for molecular analysis, one portion was fixed in 4% paraformaldehyde for FISH and Raman confocal microscopy analysis, and the other portion was fixed in 2.5% (vol/vol) glutaraldehyde and 4.0% (vol/vol) paraformaldehyde solutions for electronic microscopy (EM) analyses. The dissolved oxygen (DO) concentrations in the *S. crosnieri* community were determined *in situ* over 72 h using a RINKO I detector (JFE Advantech, Japan). Methane concentrations were also measured using a HydroC methane meter (CONTROS, Germany) (see Fig. 6). Environmental microorganisms were simultaneously collected using a WTS-LV instrument inside the *S. crosnieri* community. Total DNA was then extracted from the samples using an E.Z.N.A. soil DNA kit (Omega, USA). Total RNA was also extracted and purified, using the conventional TRIzol method (Invitrogen, USA). Filtered microbial community samples were also extracted, using a power-water DNA extraction kit (Qiagen, USA). Library preparation and sequencing were conducted at Novogene Co., Ltd. (Tianjin, China) using 2 × 150 paired-end sequencing on the Illumina NovaSeq 6000 platform (File S1).

### Deep-sea *in situ* experiments.

A deep-sea *in situ* incubation experiment was conducted to identify the responses of episymbionts to hypoxic conditions using a newly designed incubation device (Fig. S6). The pilot study showed that DO concentrations decreased to near 0 after 3 h of incubation in the sealed tank. Four *S. crosnieri* lobsters were placed in the sealed device. After 4 h of incubation, a solution containing methane and thiosulfate was released into the tank to achieve a final concentration of 100 μmol/L in the device. The oxygen concentrations remained below 0.5 mg/L until the end of the experiment at 12 h. The watertight incubation devices were retrieved from the deep sea after 2 h, during which the DO concentrations inside the device did not change. The seawater in the device was also sampled and stored at −20°C and used to subsequently measure variation in environmental parameters within incubation waters of the experiment. A microbore continuous-flow analyzer (SKALAR-SAN++, The Netherlands) was used to determine ammonia, nitrite, nitrate, and phosphate concentrations in seawater using standard colorimetric methods according to the manufacturer’s specifications (83). In addition, lobsters that were collected when oxygen concentrations were high were used as oxic controls. Immediately after bringing samples onboard, they were dissected and frozen in liquid nitrogen for transcriptomic analyses.

### Metagenome and metatranscriptome sequencing.

Agarose gel electrophoresis was used to check the DNA purity and integrity. A Qubit 2.0 fluorometer was used for the accurate DNA concentration measurement. Physical fractionation of the DNA was applied by a Covaris sonicator. During the fractionation steps, an Agilent 2100 instrument and quantitative PCR (qPCR) were adopted to ensure sufficient enrichment of the target fragments. Then a range of end-repairing, A-tailing, ligation of sequencing adapters, size selection, and PCR enrichment steps were done to produce the libraries. Sequencing was performed using the Illumina NovaSeq 6000 platform after library clustering with paired-end reads.

Total RNA was qualified and quantified as follows: (i) the RNA sample was first qualified using 1% agarose gel electrophoresis for possible contamination and degradation; (ii) RNA purity and concentration were then examined using a NanoPhotometer spectrophotometer; (iii) the RNA sample was precisely qualified with a Qubit 2.0 fluorometer; (iv) RNA integrity and quantity were finally measured using the RNA Nano 6000 assay kit of the Bioanalyzer 2100 system. The RNA library was prepared following the rRNA depletion method. Briefly, the rRNA was depleted from the total RNA using the rRNA removal kit following the manufacturer’s instructions. RNA was then fragmented into 250- to ~300-bp fragments and subsequently reverse-transcribed into cDNA. The remaining overhangs of double-strand cDNA were converted into blunt ends via exonuclease/polymerase activities. After adenylation of 3′ ends of DNA fragments, sequencing adaptors were ligated to the cDNA. In order to select cDNA fragments of preferentially 250 to ~300 bp in length, the library fragments were purified with the AMPure XP system. Amplification of cDNA was performed using PCR. After library construction, the concentration of the library was measured using the Qubit fluorometer and adjusted to 1 ng/μL. An Agilent 2100 Bioanalyzer was deployed to examine the insert size of the acquired library. Last, the accurate concentration of cDNA library was again examined using qPCR. Once the insert size and concentration of the library were identical, the samples could then be subjected to sequencing.

### Bioinformatics analyses.

Low-quality reads were trimmed and filtered using Fastp (84). phyloFlash was then used to perform RNA small subunit (SSU) screening, reconstruction, and qualification. SSU-based phylogenetic trees were constructed using the maximum-likelihood model in MEGA (v10.2) (85) with default parameters.

Quality-controlled clean metagenomic data were assembled using metaSPAdes (v3.1) with kmer sizes of 21, 33, 55, 71, 91, and 101 (Fig. 1D) (86). The long contigs (above 1 kbp) were further binned into MAGs using the MaxBin2 (v2.2.7), MetaBat2 (v2.12.1), CONCOCT (v1.1.0), VAMB (v3.0.1), SolidBin (v1.3), and BinSanity (v0.5.4) binning programs. Binning results were then refined using MetaWRAP (v1.3) based on quality metrics implemented in the CheckM software package (v1.1.3) (87). MAGs were reassembled to obtain the highest-quality MAGs using metaSPAdes (v3.14.1), as implemented in the MetaWRAP pipeline. MAG redundancies were then minimized using the dRep program (v2.5.4) (88). The final MAGs were annotated with the NCBI Prokaryotic Genome Annotation Pipeline (PGAP). Genes were also compared against the NCBI nonredundant (nr) database, the Kyoto Encyclopedia of Genes and Genomes (KEGG) database, and the Protein Families (Pfam) Database. In addition, gene ontology (GO) functional mappings, as implemented in the OmicBox software program, were assigned using the nr annotations. Each KEGG module’s completeness was determined with an in-house python script (available in GitHub, see “Data Availability”).

The taxonomic classification of each MAG was determined using the Genome Taxonomy Database Toolkit (GTDB-Tk) (v1.4.0) and further confirmed with phylogenetic analysis. Orthologous proteins were determined with OrthoFinder (v2.5.1) (89) and aligned with PRANK (90), followed by trimming with TrimAl (91). The final data set was used to construct separate gene trees with RAxML and the CAT+GTR substitution model. Finally, a species tree was constructed from the best-scoring ML trees for each gene using the ASTRAL-MP program (92).

Metatranscriptomic analysis was conducted using the quality-controlled clean data that was aligned against MAGs using the MagicBlast program and a percent identity cutoff of 98%. A transcriptome sequencing (RNA-seq) count matrix was generated using the featureCounts program (93). The expression levels of genes were then quantified using the fragments per kilobase per million mapped fragments (FPKM) metric with the FPKM_count.py function of the RSeQC package. Other statistical analyses, such as nonparametric ANOVA tests, were conducted in R (v4.0).

### Fluorescence *in situ* hybridization (FISH).

FISH imaging was used to investigate the distribution of dominant episymbionts, as previously described (94). Briefly, taxon-specific FISH probes were designed based on recovered 16S rRNA gene sequences (Fig. S7). A previously published probe (EPSY549) was also used for the *Campylobacterota*. Paraffin sections with 6-μm width were dewaxed and rehydrated in xylene and graded alcohol solutions (100%, 90%, 80%, 70%, and 50%), followed by incubation with proteinase K (10 μg/mL, pH 8) and lysozyme (5 μg/mL, pH 8) solutions for 0.5 h to initiate permeabilization. The slides were then serially dehydrated in an ethanol gradient (70%, 80%, 90%, 95%, and 100%) and dried at 46°C for 3 h. Slides were hybridized for 3 h at 46°C in hybridization buffer (0.9 M NaCl, 0.02 M Tris-HCl, 0.01% SDS, and 30% formamide) with probes (10 ng/μL). Negative controls were consistently implemented with the NonPolyPr350 polynucleotide probe. The slides were finally washed in washing buffer (0.1 M NaCl, 0.02 M Tris-HCl, 0.001% SDS, and 5 mM EDTA) for 15 min at 48°C and then mounted and observed on an LSM 900 confocal laser scanning microscope (Zeiss, Germany).

10.1128/msystems.00320-22.7 FIG S7 (A and B) Phylogeny of the symbiont 16S rRNA genes (A) and taxa-specific FISH probes (B). Download FIG S7, TIF file, 0.4 MB. Copyright © 2022 Xu et al. 2022 Xu et al. https://creativecommons.org/licenses/by/4.0/ This content is distributed under the terms of the Creative Commons Attribution 4.0 International license.

### Electronic microscopy (EM) observations.

For EM, samples were dehydrated in a graded ethanol series, critical point dehydrated, and then coated with gold (sputter/carbon thread, EM ACE200). Prepared samples were then observed with scanning EM (VEGA3, TESCAN). For TEM analysis, fixed samples were postfixed in 2% (wt/vol) aqueous osmium tetroxide (Electron Microscopy Sciences). Samples were then rinsed, dehydrated, and embedded in Ep812 resin. Ultrathin sections were cut with an ultramicrotome at a thickness of 70 nm (Reichert-Jung Ultracut E). Sections were then double-stained with lead citrate and uranyl acetate, followed by slide observation with a TEM (JEM1200, JEOL) operated at 100 KV.

### Raman spectroscopy.

To detect and localize sulfur in the episymbiont cells, Raman imaging of setae was conducted with a confocal Raman spectrometer (Alpha 300R+, WITec, Ulm, Germany) using a 50×/0.75 lens (Zeiss, EC, Epiplan-Neofluar, Germany) with a 532-nm laser as the light source, set with a laser power of 15 mW. Three Raman images were measured in sequence using a 10-s integration time at a 2-μm spatial resolution. Before the experiment, silicon was used for laser wavelength calibration and verification based on its spectral line.

### Data availability.

The metagenomic and metatranscriptomic raw reads are available in the NCBI database under the BioProject accession number PRJNA728519. All scripts used in the manuscript are available in GitHub (https://github.com/lobstar1/KEGG-module-completeness).
