# Supplementary figures and images for: Metabolism Interactions Promote the Overall Functioning of the Episymbiotic Chemosynthetic Community of Shinkaia crosnieri of Cold Seeps
Source: mSystems. 2022 Aug 8;7(4):e00320-22. doi: 10.1128/msystems.00320-22 (PMC9426478; doi:10.1128/msystems.00320-22)

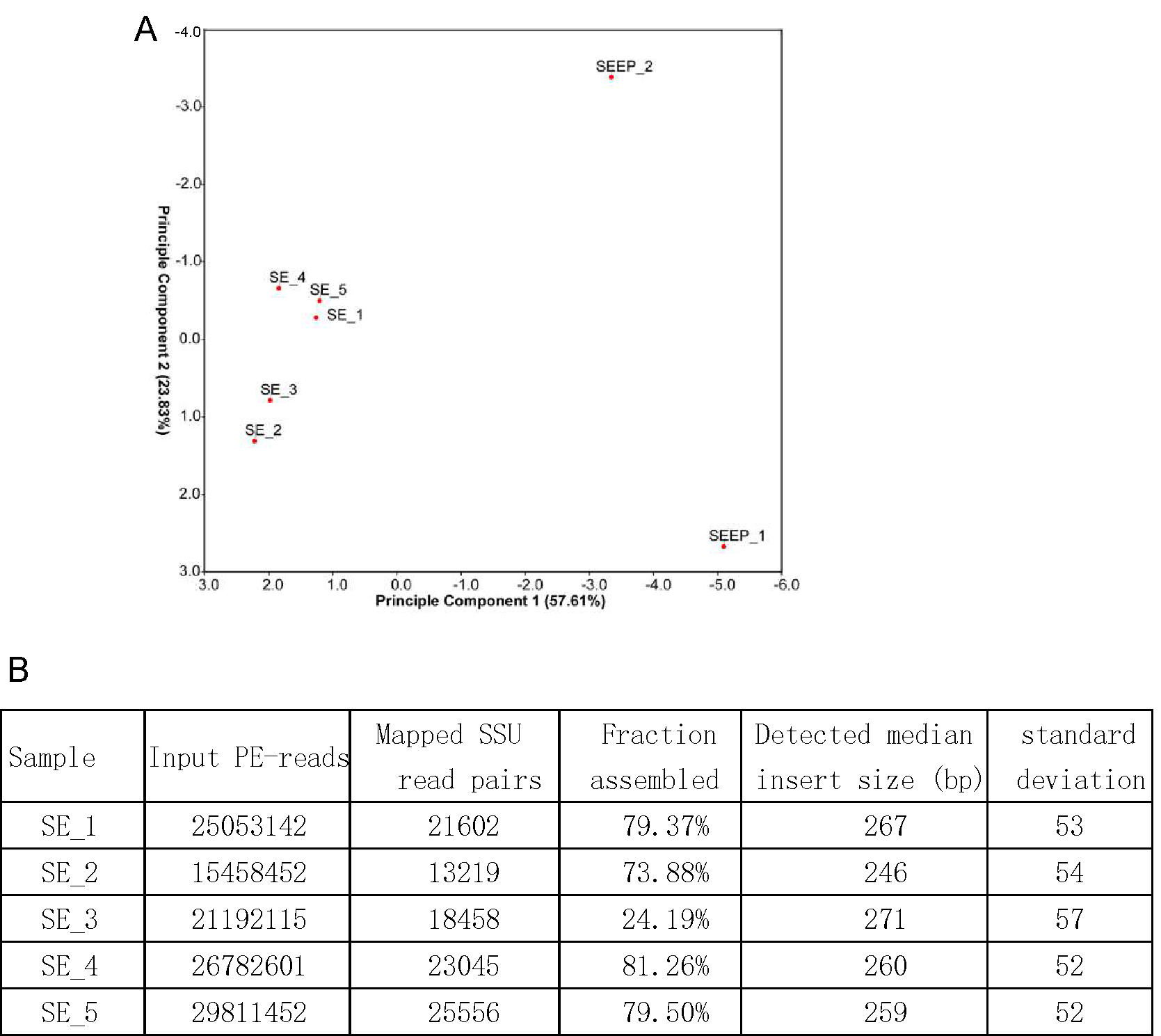

Supplement: FIG S1 [file msystems.00320-22-s0001.tif]

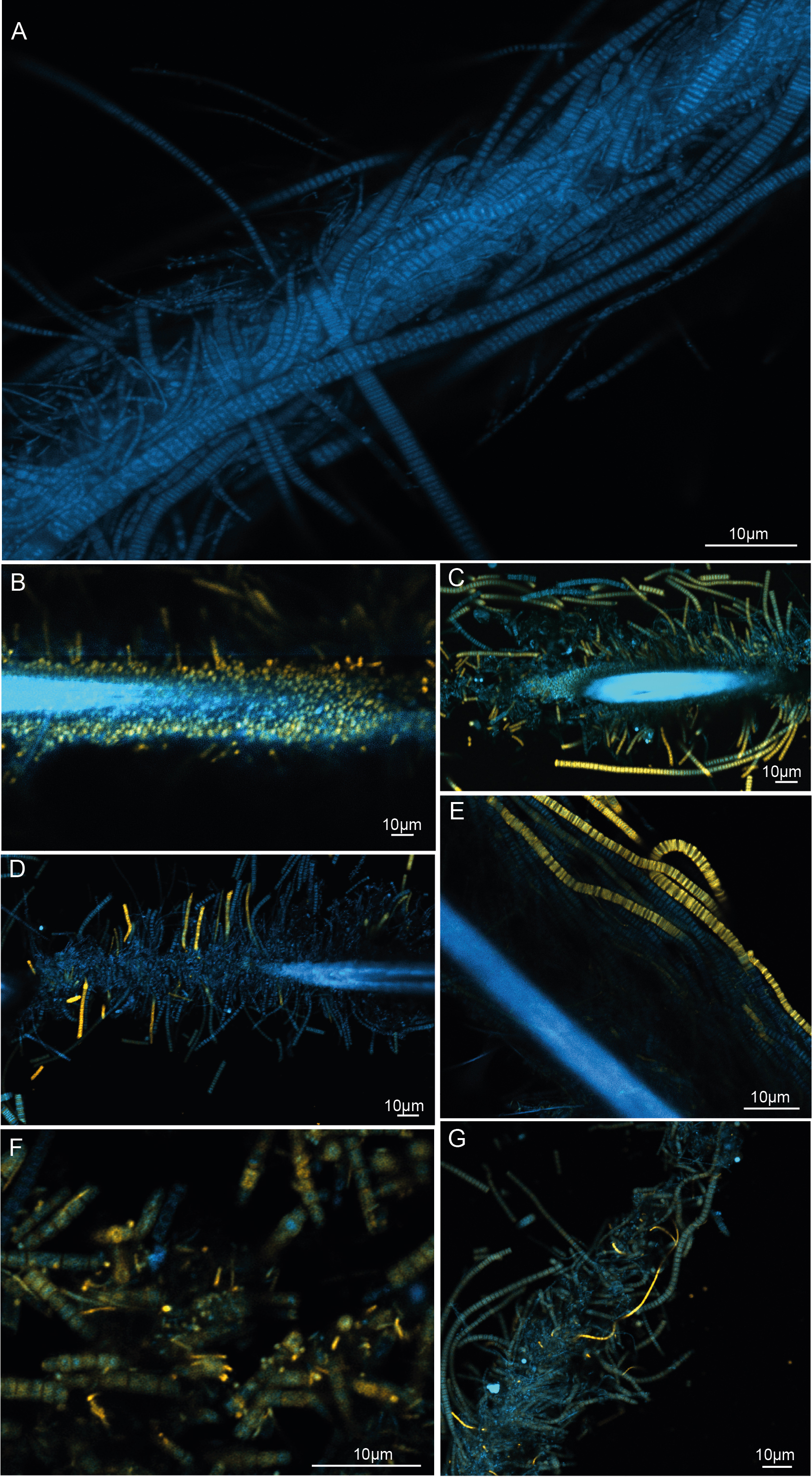

Supplement: FIG S2 [file msystems.00320-22-s0002.jpg]

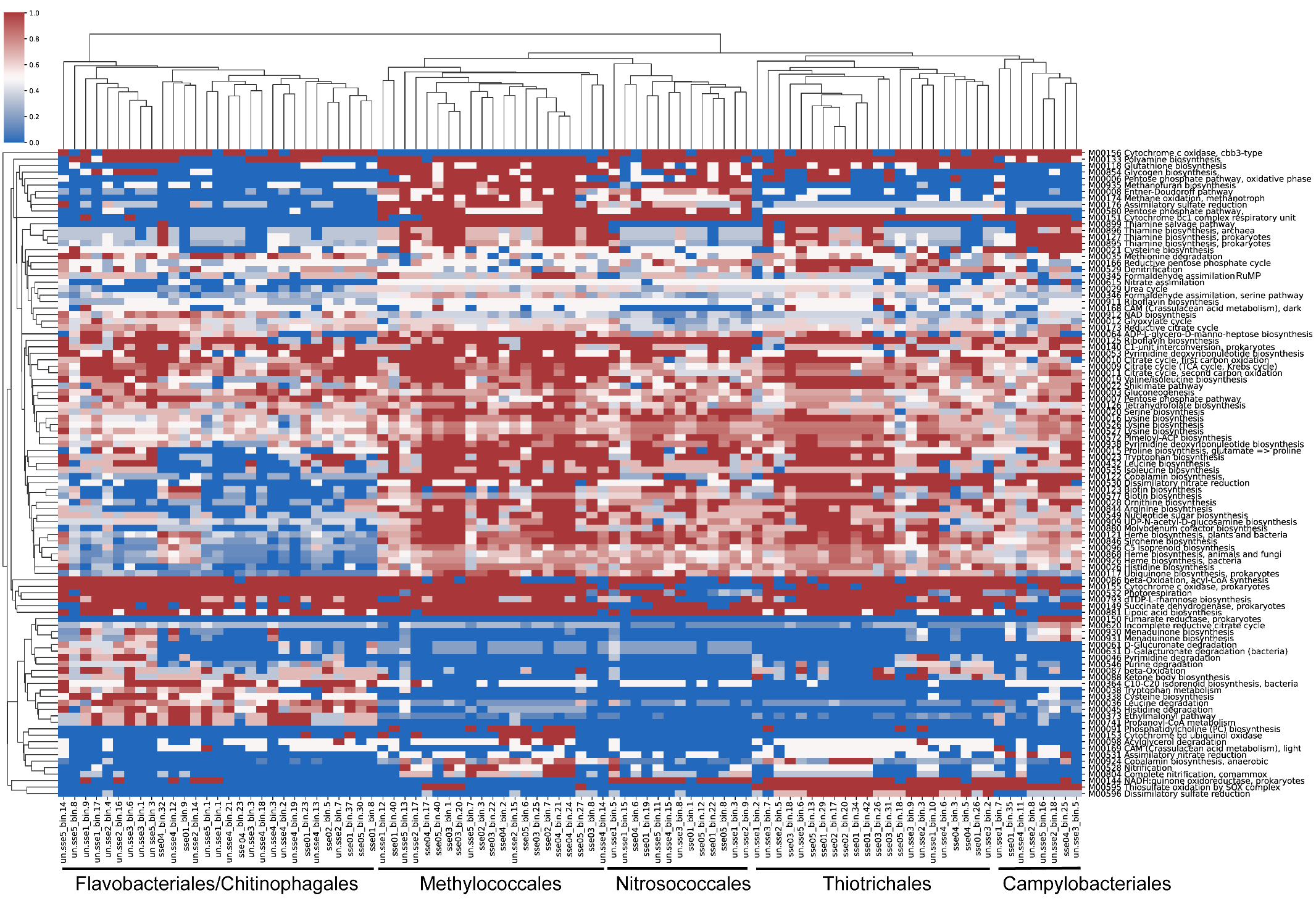

Supplement: FIG S3 [file msystems.00320-22-s0003.tif]

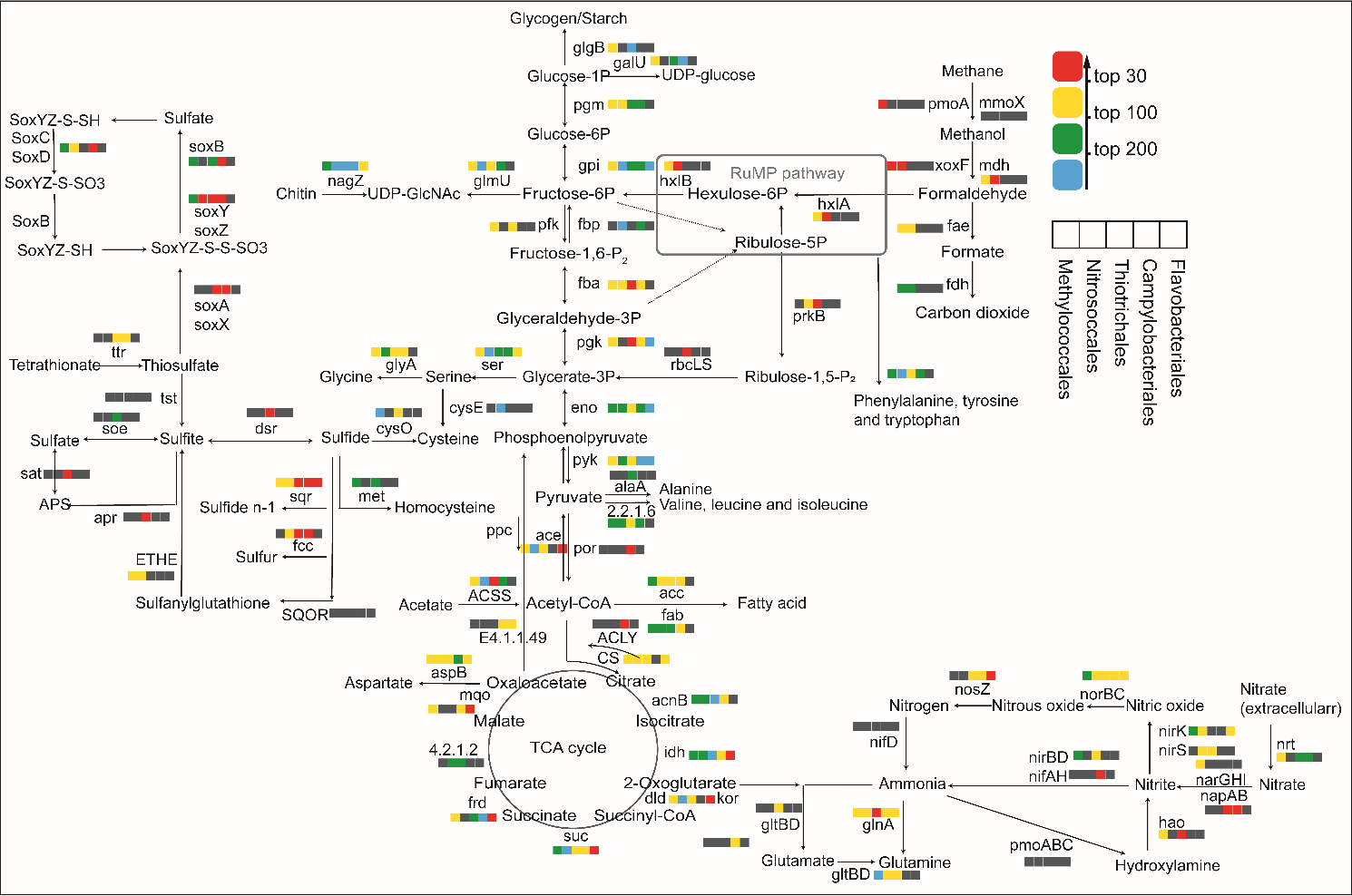

Supplement: FIG S4 [file msystems.00320-22-s0004.tif]

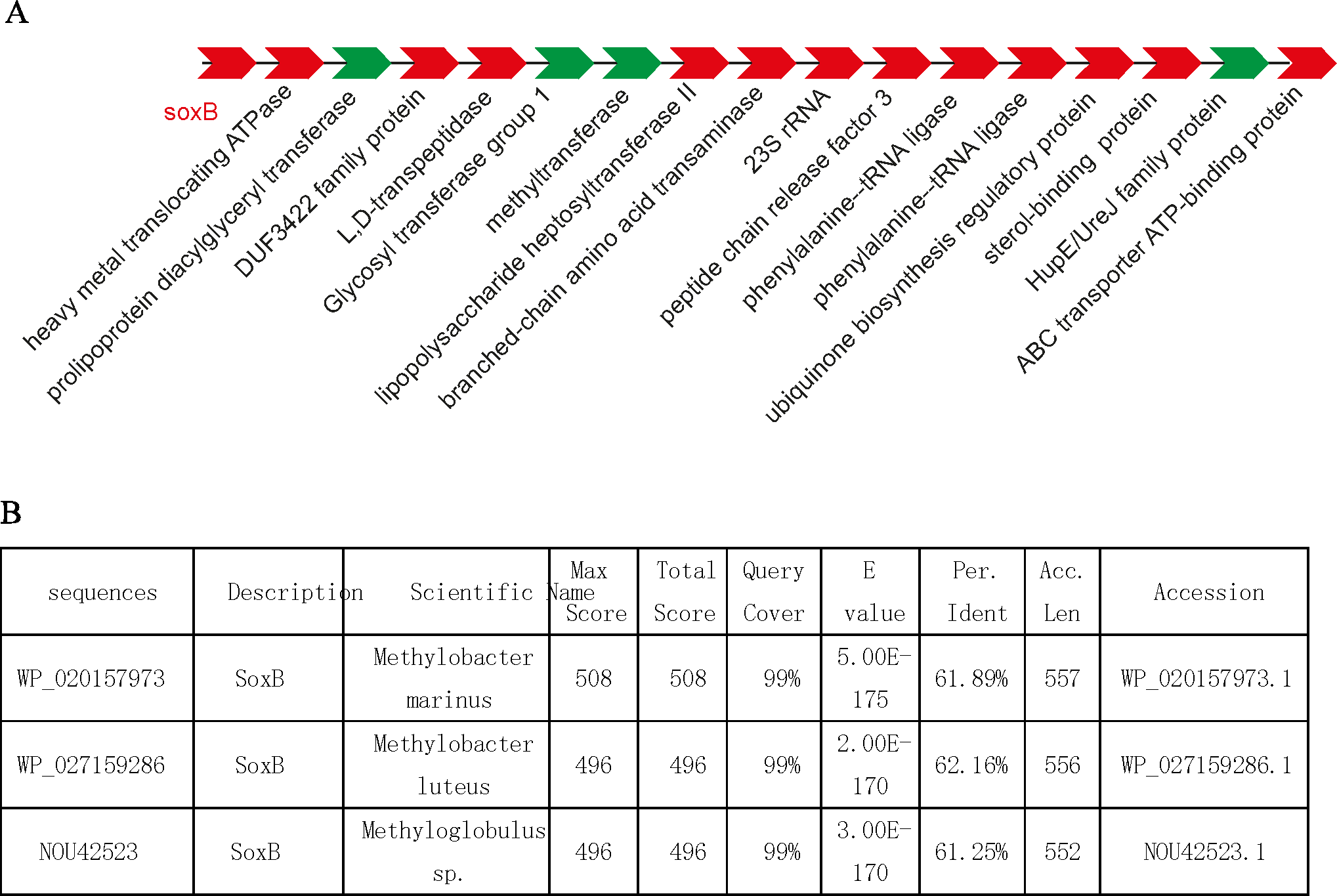

Supplement: FIG S5 [file msystems.00320-22-s0005.tif]

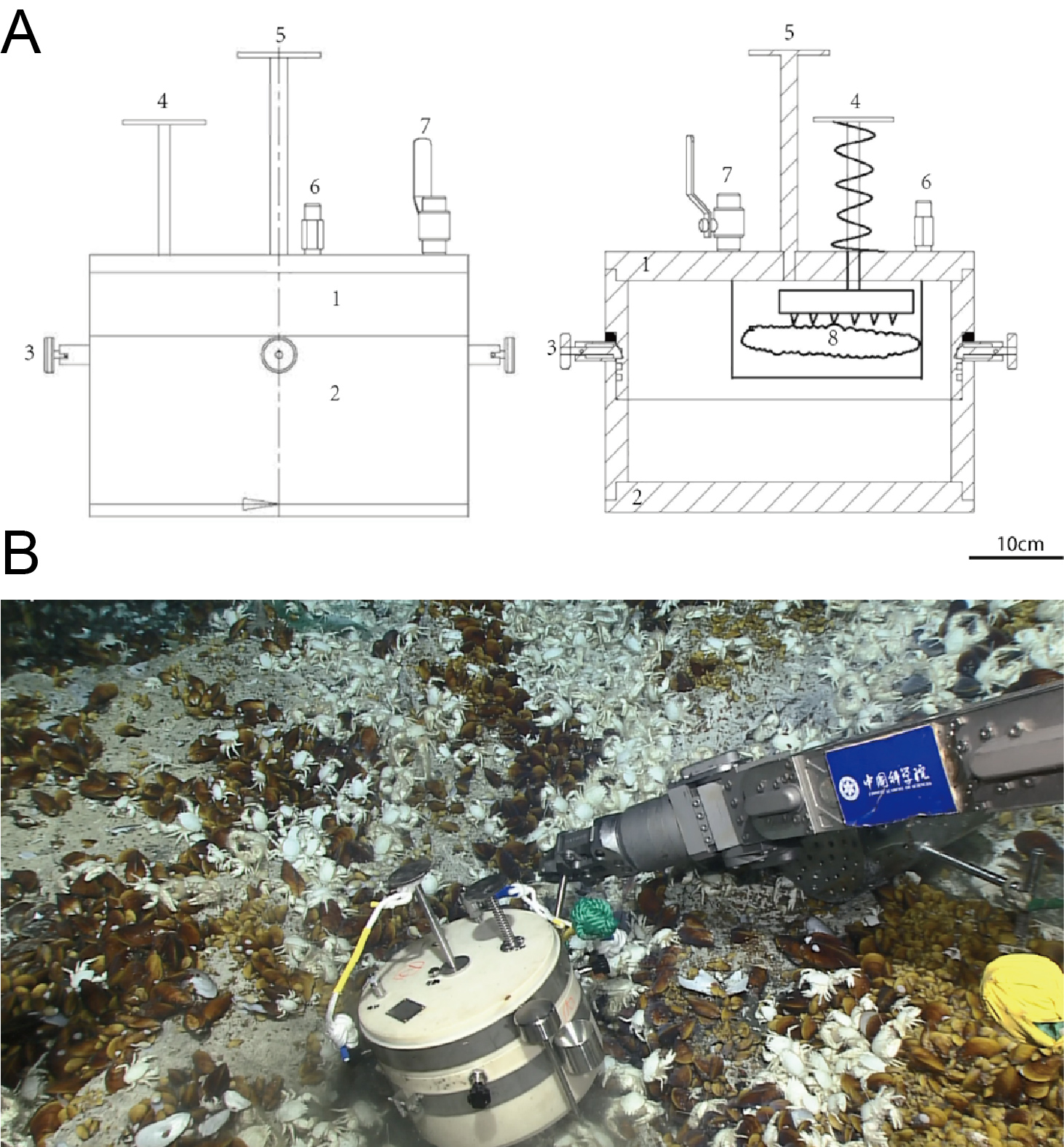

Supplement: FIG S6 [file msystems.00320-22-s0006.jpg]

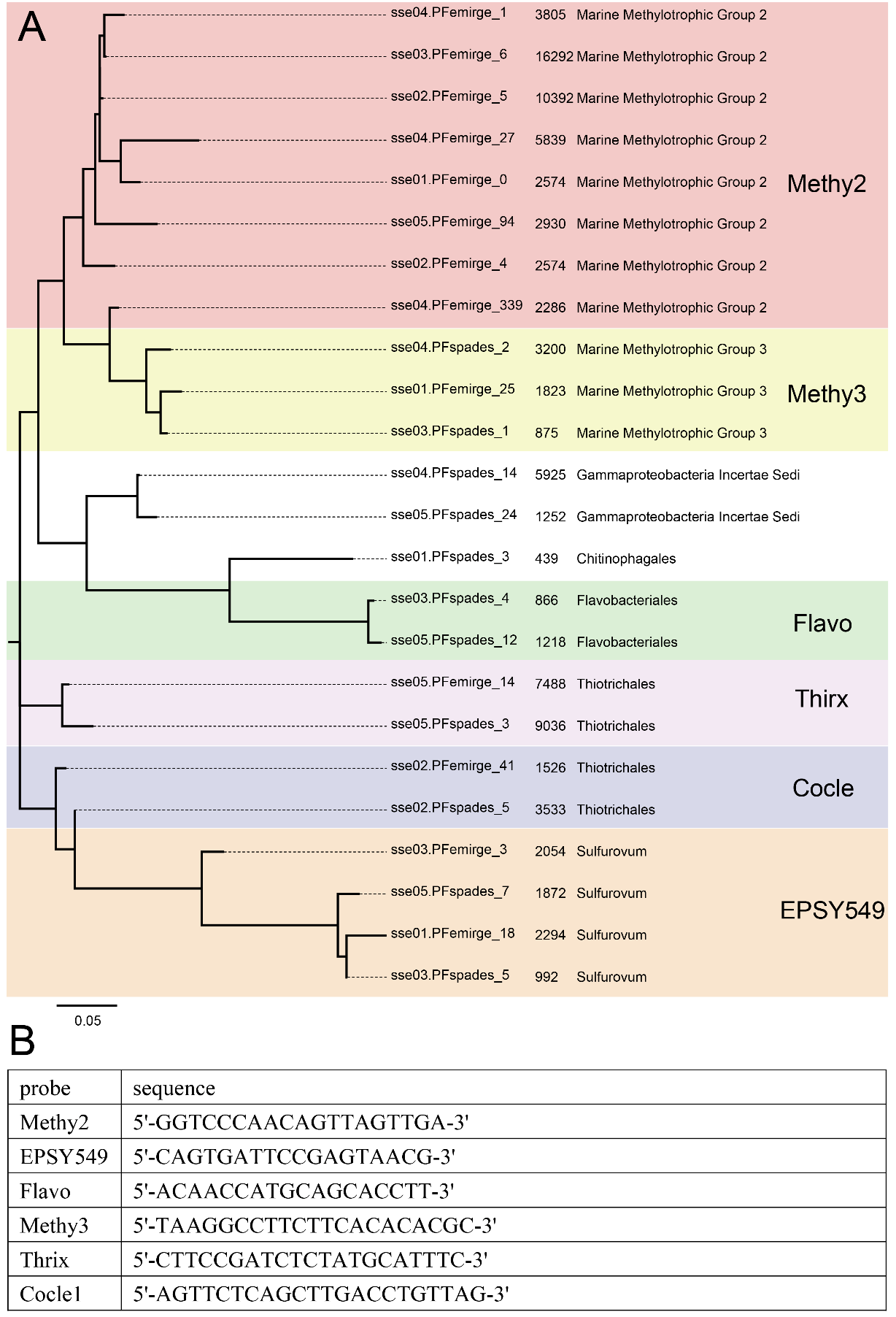

Supplement: FIG S7 [file msystems.00320-22-s0007.tif]
